# Complete Genomic Sequence Analysis of a Sugarcane Streak Mosaic Virus Isolate from Yunnan Province of China

**DOI:** 10.3390/genes14091713

**Published:** 2023-08-28

**Authors:** Xiao-Ling Su, Zhong-Yue Mai, Kun-Jiang Wei, Yang-Jian Huang, Hong-Li Shan, De-Jie Cheng

**Affiliations:** 1 Guangxi Key Laboratory of Agro-Environment and Agric-Products Safety, Agricultural College, Guangxi University, Nanning 520004, China; 2217304027@st.gxu.edu.cn (X.-L.S.); maizy6@163.com (Z.-Y.M.); weikunjiangsyx@163.com (K.-J.W.); hyj6131@163.com (Y.-J.H.); 2 Yunnan Key Laboratory of Sugarcane Genetic Improvement, Sugarcane Research Institute, Yunnan Academy of Agricultural Sciences, Kaiyuan 661699, China

**Keywords:** sugarcane streak mosaic virus, phylogenetic analysis, recombination analysis, selective pressure analysis

## Abstract

In recent years, the sugarcane streak mosaic virus (SCSMV) has been the primary pathogen of sugarcane mosaic disease in southern China. In this study, the complete genome of a sugarcane mosaic sample (named YN-21) from Kaiyuan City, Yunnan Province, was amplified and sequenced. By comparing the amino acid sequences of YN-21 and 15 other SCSMV isolates from the NCBI database, the protease recognition site of SCSMV was determined. YN-21 had the highest nucleotide and amino acid identities of 97.66% and 99.30%, respectively, in comparison with the SCSMV isolate (JF488066). The P1 had the highest variability of 83.38–99.72% in the amino acid sequence, and 6K2 was the most conserved, with 97.92–100% amino acid sequence identity. A phylogenetic analysis of nucleotide and amino acid sequences clustered the 16 SCSMV isolates into two groups. All the Chinese isolates were clustered into the same group, and YN-21 was closely related to the Yunnan and Hainan isolates in China. Recombination analysis showed no major recombination sites in YN-21. Selective pressure analysis showed that the d_N_/d_S_ values of 11 proteins of SCSMV were less than 1, all of which were undergoing negative selection. These results can provide practical guidance for monitoring SCSMV epidemics and genetics.

## 1. Introduction

Sugarcane streak mosaic virus (SCSMV; species *Sugarcane streak mosaic virus*, genus *Poacevirus*, family *Potyviridae*) has been the primary pathogen of sugarcane mosaic disease in southern China in recent years [1,2]. SCSMV infection causes continuous or discontinuous chlorotic stripes in sugarcane leaves, decreasing cane tonnage and saccharose yield [3]. The intensity of symptoms varies with the variety of race [4]. In addition to infecting sugarcane, SCSMV can be artificially inoculated into sorghum, corn, crowfoot grass, and Sudan grass of the *Gramineae* family [3,5]. SCSMV can be transmitted mechanically or through stem cuttings over long distances, and no insect vector has been found to transmit SCSMV [3]. A high incidence of SCSMV has been reported in sugarcane-producing areas such as China, India, Thailand, and Indonesia [3,6,7,8] and has also been reported in Iran and Africa [9,10]. Currently, the management of SCSMV involves growing virus-free planting materials and disease-resistant sugarcane varieties [11].

SCSMV is a curved filamentous 890 × 15 nm particle encapsidating a single-stranded positive-sense RNA about 10 kb nucleotides in length [12]. The SCSMV genome is translated into a polyprotein and hydrolyzed by the viral-encoded proteases into 11 mature functional proteins. These include the first protein (P1), helper component proteinase (HC-Pro), the third protein (P3), the first 6K protein (6K1), cylindrical inclusion protein (CI), the second 6K protein (6K2), viral protein-linked genome (VPg), nuclear inclusion protein a-proteinase (NIa-Pro), nuclear inclusion protein b (NIb), coat protein (CP) [13,14], and P3 N-terminal fused with Pretty Interesting Potyviridae ORF (P3N-PIPO) is produced by transcriptional sliding [15].

The population structure of viruses depends on the environment, mainly geographical and host factors [16]. Gillaspie et al. isolated SCSMV from sugarcane germplasm introduced to the US from Pakistan in 1978 [17]. Then, Hema et al. obtained an SCSMV isolate from India and found that the isolate was similar to the Pakistani isolate in nucleotide identity of about 93.6% in 3′ ends and considered that the virus was a new genus in the *Potyviridae* family [12,18]. In a study of SCSMV, Wang et al. found that the population structure of Chinese isolates had no heritable difference but was different from that of other countries based on the *CP* gene [19]. Zhang et al. found that the Chinese isolates clustered into one group, showing prominent geographical characteristics [20]. Based on the *HC-Pro* gene of SCSMV, Bagyalakshmi et al. found a large difference in the variability of the nucleotide (nt) and amino acid (aa) sequences between Indian isolates and those from other Asian countries [21]. Although the genetic characteristics of SCSMV have been previously reported in Yunnan province, China, these studies only focus on the *P1*, *CP*, *HC-Pro*, and *NIa-Pro* genes of SCSMV. There are no reports on the genetic variation within the complete genome of SCSMV Yunnan isolates in China.

In this study, we collected the sugarcane sample with SCSMV (named YN-21) from the Sugarcane Research Institute, Yunnan Academy of Agricultural Sciences in Kaiyuan City, Yunnan Province, and obtained the full genome sequence of YN-21, which was compared with other SCSMV sequences deposited in GenBank, and the genome structure and codon usage bias of SCSMV was analyzed. Phylogenetic analyses of genetic diversity and population composition were performed based on whole-genome sequences. The study provides a background for the in-depth study of the virus’s origin, occurrence, and evolution and the formulation of appropriate prevention and control of the virus. 

## 2. Materials and Methods

### 2.1. Plant Material, Extraction and RT-PCR

Sugarcane (race ROC22) leaves with the typical sugarcane mosaic symptoms were collected in Kaiyuan, Yunnan province, China (103°37′ E; 23°71′ N).

**Figure 1:**
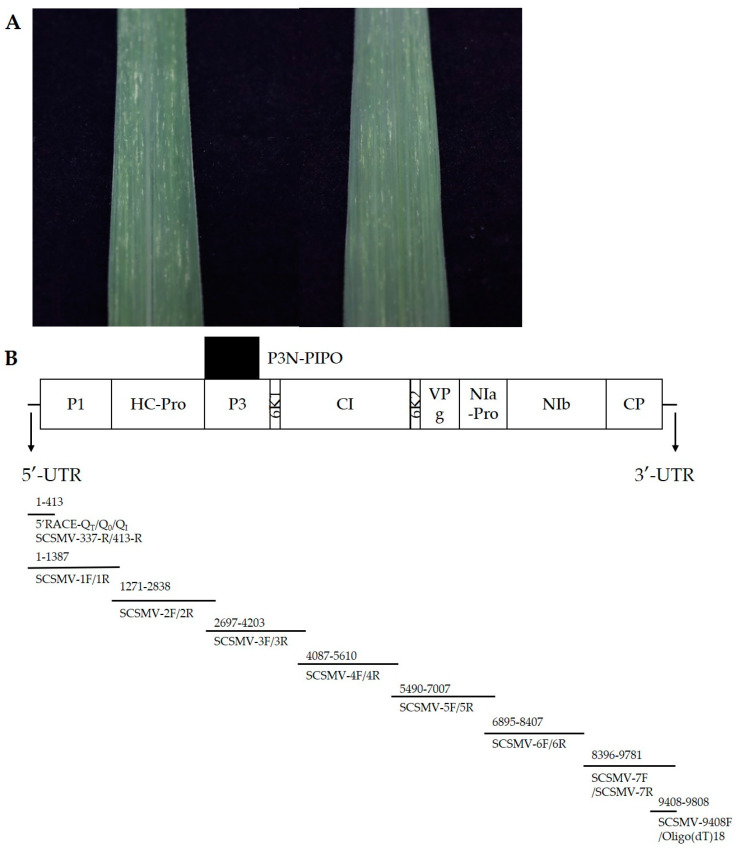
The symptoms of plants infected by SCSMV (**A**). Genome structure and amplification strategy of YN-21 (**B**).

The total RNA was extracted using Total RNA Extraction Reagent (Vazyme, Nanjing, China) according to the manufacturer’s instructions. The first strand cDNA was constructed using HiScript II 1st Strand cDNA Synthesis Kit (Vazyme, Nanjing, China). The cDNA was stored at −20 °C till further use. Using cDNA as a template and the primers designed to amplify 7 overlapping segments of the coding region of the total genome (Figure 1B) (Table A1), the short sequences of the viral genome were amplified using a 2 × taq master mix (Vazyme, Nanjing, China). The reaction conditions were as follows: initial denaturation at 95 °C for 3 min; denaturation at 95 °C for 15 s; annealing for 15 s and extension at 72 °C (extension time according to 1 kb/min) with 32 cycles; and final extension at 72 °C for 10 min. The 3′-UTR sequence was obtained using a 2 × taq master mix with the primers SCSMV-9408F and Oligo(dT)_18_. Also, the 5′-UTR sequence was amplified using the Rapid Amplification Kit for cDNA Ends (RACE) (Sangon Biotech, Shanghai, China) according to the manufacturer’s protocol. The 5′ RACE primers SCSMV-413-R and SCSMV-337-R were designed according to the isolate YN-21 5′ terminal nucleotide sequence obtained from 1F/1R. After PCR amplification, 5 μL each of the PCR products was resolved in 1% agarose gel to observe the target bands, and the PCR products were sent to Sangon Biotech (Shanghai) for sequencing. The complete genome of YN-21 was obtained by sequence assembly. 

### 2.2. Sequence Analysis

#### 2.2.1. Sequence Assembly and Determination of Protease Cleavage Sites

In order to obtain the complete genome sequence of YN-21, we used DNAMAN V6.0.3.99 and SeqMan program in DNASTAR V11.1 software to assemble the sequence of the fragments [22]. Complete genome sequences of 15 SCSMV isolates were obtained from the NCBI (National Center for Biotechnology Information, https://www.ncbi.nlm.nih.gov/ (accessed on 25 September 2022)) database, and aligned together with the YN-21 genome sequence using the DNAMAN program and the online analysis database EMBL-EBI (https://www.ebi.ac.uk/ (accessed on 31 July 2023)). According to the protease digestion site of *Potyviridae* described by Adams et al., we reclassified the protease digestion sites of SCSMV [13,15,23,24].

#### 2.2.2. Recombination Analysis, Phylogenetic and Genetic Distance Analysis

Recombination analysis was performed using RDP [25], GENECONV [26], BOOTSCAN [27], MAXCHI [28], CHIMAERA [29], 3SEQ, and SISCAN [30] algorithms in the RDP4 software. Each software parameter adopted the default value, and the *p*-value was 0.05 [31]. When more than 4 kinds of algorithms supported recombination, and *p* < 1.0 × 10**^−^**^6^, the fragment was considered to have clear recombination, otherwise it was considered that there was no recombination. To check the presence of recombination breakpoints in the analyzed data, the Genetic Algorithm for Recombination Detection (GARD) method, as implemented in the Datamonkey server (http://www.datamonkey.org/ (accessed on 28 September 2022)), was used.

#### 2.2.3. Phylogenetic and Genetic Distance Analysis

The Clustal W program in MEGA v11.0 software was used to align the nucleotide sequences of 16 SCSMV isolates, including YN-21 [32]. All phylogenetic trees were generated by the maximum likelihood method with 1000 bootstraps [33]. Maize dwarf mosaic virus (MDMV, AJ001691) was included as the outgroup. Meanwhile, the Kimura two-parameter model (K2P) in MEGA v11.0 was used to calculate the genetic distance between and within groups.

#### 2.2.4. Selection Pressure Analysis

Selection pressure on each viral gene was predicted by calculating the d_N_/d_S_ (non-synonymous/synonymous) value between different genes. The codon selection pressure of the 11 genes in the 16 SCSMV isolates was determined by FEL [34] in the online analysis software Datamonkey, and the *p*-value was 0.1. Values of d_N_/d_S_ < 1, d_N_/d_S_ = 1, and d_N_/d_S_ > 1 indicated negative (or purifying) selection, neutral evolution, and positive (or diversifying) selection, respectively.

## 3. Results

### 3.1. Genome Base Composition and Codon Preference of YN-21

Sugarcane (race ROC22) leaves showing the typical sugarcane mosaic symptoms were collected in the Yunnan province of China (Figure 1A). 

The total length of the YN-21 (GenBank number OR259188) genome is 9808 nucleotides and the coding sequence is 9390 nucleotides which encodes 3130 amino acids (Table A2). Within the polyprotein, leucine (Leu) was the most abundant, with 264, accounting for 8.43%, while cysteine (Cys) was the least abundant, with 51, accounting for 1.63%.

**Figure 2:**
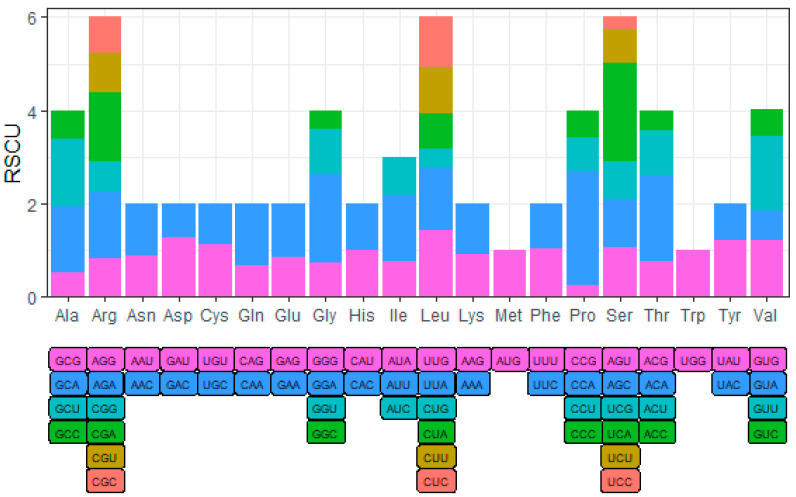
The RSCU value of codons in YN-21. RSCU > 1, the codon is relatively frequently used.

In order to explore the preference of YN-21 for different codons in the process of translation, we analyzed the relative synonymous codon usage (RSCU). When the RSCU value of the codon is greater than 1, it means that the codon is a relatively frequently used codon. We found that the codons of all amino acids showed preference, except tryptophan (Trp) and methionine (Met): GAU (Asp); GAA (Glu); UUA, CUC, and UUG (Leu); AUU (Ile); GUU and GUG (Val); UCA, AGU, and AGC (Ser); CCA (Pro); ACA (Thr); GCU and GCA (Ala); UAU (Tyr); CAA (Gln); GGA (Gly); UGU (Cys); UUU (Phe); CAU (His); AAC (Asn); AGA and CGA (Arg) (Figure 2) (Table A2).

### 3.2. YN-21 Protease Recognition Site Analysis

The YN-21 polyprotein is cleaved by its own protease into 11 mature proteins, respectively, P1 (358 aa, 41.51 kDa), HC-Pro (470 aa, 54.37 kDa), P3 (330 aa, 37.42 kDa), 6K1 (49 aa, 5.43 kDa), CI (656 aa, 74.81 kDa), 6K2 (48 aa, 5.55 kDa), VPg (198 aa, 22.46 kDa), NIa-Pro (238 aa, 26.63 kDa), NIb (502 aa, 57.44 kDa), CP (281 aa, 31.05 kDa), and P3N-PIPO (274 aa, 32.06 kDa), produced by transcriptional sliding (Table 1).

### 3.3. YN-21 Concordance Rate with 15 SCSMV Isolates

The sequence alignment between YN-21 and the coding sequences of 15 SCSMV isolates from the GenBank database showed that YN-21 had the highest nucleotide and amino acid percentage identities of 97.66% and 99.30% with ID (Accession number JF488066), respectively. By analyzing the concordance rate of 11 proteins in 16 sequences, we found the greatest variation in the P1 with 83.38–99.72% amino acid sequence identities, and 6K2 was the most conserved protein with 97.92–100% amino acid sequence identities. 

By comparing the nucleotide sequences of SCSMV isolates, the percentage identities between YN-21 and the other 15 isolates were high, up to 96.5%. Of these, the highest identity rates of the 11 genes within the SCSMV isolates is 85.88–97.98% of *CP* nucleotide sequences. At the genome level, the nucleotide and amino acid sequence identities between YN-21 and the other 15 SCSMV isolates were 80.98–97.66% and 92.12–99.30%, respectively. Among the 15 isolates, isolate JF488066 had the highest nucleotide and amino acid sequence identities with isolate YN-21 (Table A3).

### 3.4. Recombination Analysis

The analysis performed with RDP4 revealed the presence of recombination breakpoints in 7 of 16 SCSMV isolates (Table 2). Meanwhile, the recombination analysis of the coding sequences (CDS) of 11 genes showed that there were no obvious recombination sites in *6K1*, *6K2*, and *VPg*. Three recombination regions were found in *CI*, and one each was found in *CP* and *P1*, respectively (Table 3). The CDS with recombinant sites belonged to cluster group II, and no recombinant was found in group I, indicating that the frequency of recombination was low among SCSMV isolates from China. In order to test the reliability of recombination, GARD was used for recombination analysis, and the results of GRAD were found to be consistent with those of RDP4.

**Figure 3:**
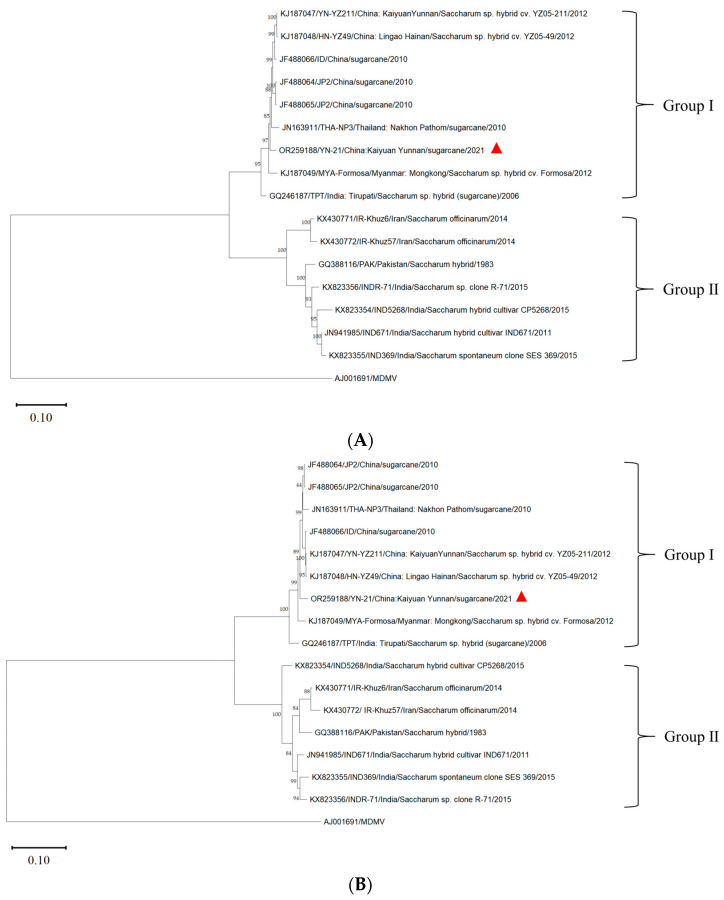
Phylogenetic tree of two partitions of SCSMV genomes obtained after deleting recombinant genes of YN-21 (triangle symbol) and 15 SCSMV isolates. The MDMV was used as an outgroup. The bootstrap value was 1000. Phylogenetic tree including *HC-Pro*, *P3*, and *6K1* gene sequences (**A**). Phylogenetic tree including *6K2*, *VPg*, *NIa-Pro*, and *NIb* gene sequences. (**B**).

In order to reduce the impact of recombination on the population structure, we excluded the three genes that have recombination and divided the SCSMV genome into two segments, A (*HC-Pro*, *P3*, *6K1*) and B (*6K2*, *VPg*, *NIa-Pro*, *NIb*), using the maximum likelihood method to construct a phylogenetic tree with a bootstrap value of 1000 (Figure 3).

### 3.5. Phylogenetic Analysis

**Figure 4:**
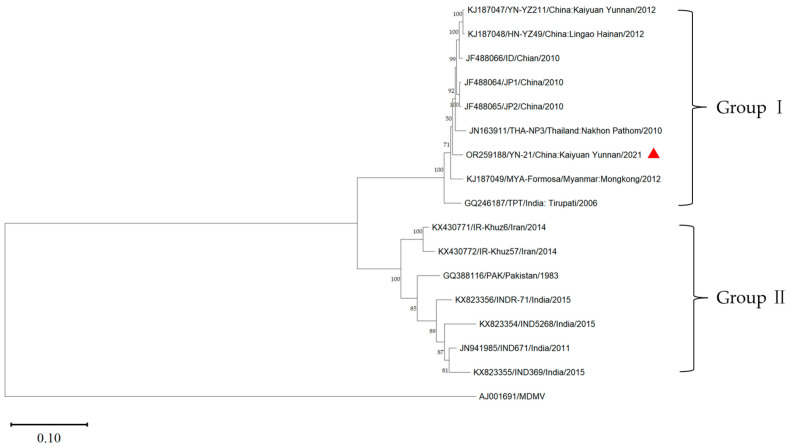
Maximum likelihood tree calculated from the complete genome of YN-21 (triangle symbol) and 15 SCSMV isolates. The MDMV was used as an outgroup. The terminal node consists of the accession number, isolate name, collection location, and time.

Using the MDMV (AJ001961) genome as the outgroup, the phylogenetic tree of the complete genome of YN-21 and 15 other isolates was constructed by the maximum likelihood method using the software MEGA v11.0 (Figure 4). The sixteen SCSMV isolates were clustered into two groups (group I and group II). Group I included YN-21 and eight isolates from Yunnan (YN-21211, ID, JP1, JP2) and Hainan (HN-YZ49) in China, Thailand (THA-NP3), Myanmar (MYA-Formasa), and India (TPT). Group II included seven isolates from Iran (IR-Khuz57, IR-Khuz6), Pakistan (PAK), and India (IND5268, INDR-71, IND369, IND671). The results indicated that the YN-21 isolate was closely related to the SCSMV isolates from the Yunnan and Hainan provinces in China. We found that the grouping of the phylogenetic tree without recombinant genes is consistent with that of the full genome, but there are small differences between the second partition and the full genome.

To verify the reliability of the grouping results, we further calculated the genetic distance between and within groups (Table 4). The average genetic distances of group I and group II were 0.03 and 0.10. The genetic distances within the group were lower than the genetic distances between groups, indicating that the results of the phylogenetic analysis are reliable.

### 3.6. Selection Pressure Analysis

The d_N_/d_S_ value of different populations of SCSMV was less than 1, and there was one positive selection site in each of P1 (322nd codon) and P3N-PIPO (240nd codon) (Table 5). The d_N_/d_S_ value of 0.112 for P3N-PIPO was the highest, and the d_N_/d_S_ value of 0.0013 for 6K2 was the lowest, showing that different populations of SCSMV were under strong negative selection pressure.

## 4. Discussion

In a previous study, Xu et al. compared the sequence of PAK (GQ388116) with other representative species of the *Potyviridae* family and obtained the polyprotein cleavage site of SCSMV [1]. In this study, we compared the complete genome sequences of 16 SCSMV isolates and found that the conserved positions of amino acids were different from the results of Xu et al.—the amino acids at the cleavage site are more conservative, which may be because the comparison may only be made in the same species—and deduced the cleavage site of P3N-PIPO. Based on the results of gene RSCU analysis, we found that 25 codons in YN-21 had RSCU values greater than 1, of which 11 codons ended in A and 9 codons ended in U, indicating that YN-21 prefers codons ending in A and U. Codon translation rates are positively correlated with its usage frequency and receptor tRNA concentration [35]. Therefore, we believe that the codon bias of YN-21 exists as a result of its evolution toward higher rates of gene expression, and may also be influenced by tRNA abundance. In other words, YN-21 has evolved to continuously select codons that produce proteins rapidly, thereby increasing the growth rate of the virus.

Recombination occurs frequently in *Potyviridae*, which can improve the adaptive evolution of RNA virus to the host, break host resistance, and produce virus strains suitable for epidemic [36,37]. Previous studies have shown that the *HC-Pro* gene of Indian SCSMV isolate showed recombination events [21], while the *CP* gene of Chinese SCSMV rarely recombined and was under negative selection pressure [20]. In this study, the coding sequence of SCSMV was analyzed by recombination analysis and selective pressure analysis. The recombination sites were only found in the *CI*, *CP*, and *P1* genes, and no obvious recombination sites were found in YN-21 isolates. The d_N_/d_S_ value among different populations of SCSMV is less than 1, but there is one positive selection site in P1 and P3N-PIPO, indicating that Chinese SCSMV isolates seldom recombine and are under negative selection pressure. This shows that recombination is not the main driving force of SCSMV molecular evolution, but negative selection is the main force driving SCSMV evolution.

The genome contains a great deal of genetic information about biological evolution. By establishing a phylogenetic tree, we can infer the genetic relationship among species and the evolutionary process of speciation [38]. Based on the phylogenetic analysis of the *P1* and *CP* genes, SCSMV populations were grouped into two groups, and all Chinese SCSMV isolates were clustered into one group [7]. The phylogenetic analysis of SCSMV *CP* genes showed obvious geographical variations, and the SCSMV isolates from China were clustered into one group [19]. However, SCSMV isolates from India, Australia, South Africa, and the United States were distributed into 14 phylogroups, implying that the virus isolates could not be simply classified according to the geographical origin of the host species [39]. Based on the phylogenetic analysis of the complete genomes of 16 SCSMV isolates, we found that the SCSMV isolates from China clustered into one group, and YN-21 was closely related to the isolates from the Yunnan and Hainan provinces of China, indicating that the population of the SCSMV isolates from China had no obvious genetic differentiation, which was consistent with the results of the complete gene sequence analysis of SCSMV by Li et al. [40]. After removing the genes with recombination sites, phylogenetic analyses reveal that the grouping of SCSMV isolates into two main phylogenetic groups is consistent with both phylogenetic trees constructed based on the full genome sequences as well as those constructed based on particular partitions. 

Some viruses from *Potyviridae* can be transmitted by aphids in a non-persistent way, such as the sugarcane mosaic virus [41] and the sorghum mosaic virus [11]. SCSMV can be transmitted mechanically by infiltration, friction inoculation, and knife injury, as well as through asexual reproduction materials such as stem cuttings. There is no evidence of insect transmission [3], possibly due to the lack of KITC, PTK, and DAG motifs in the HC-Pro and CP, which are necessary for the aphid transmission of SCSMV [1]. Germplasm exchange between different countries and the transfer of seedlings between different regions is the only path for the long-distance transmission of SCSMV. YN-21 is closely related to isolates from Thailand and Myanmar, which may be due to the frequent germplasm exchange between Yunnan province of China, Myanmar, and Thailand in recent years and the spread of SCSMV with the spread of seedlings [42].

## Figures and Tables

**Table 1:** Genomic structure of YN-21 and protease cleavage sites determined by the comparison of YN-21 with other SCSMV isolates. Conserved amino acids in the cleavage sites are bolded.

Genome Fragment	Start-End Site	Size in nt/aa	Cleavage Site (C-Terminus)
5′-UTR	1–199	199	
P1	200–1273	1074/358	**E**DL**VFY**/**T**
HC-Pro	1274–2683	1410/470	**MKYRIG**/**G**
P3	2684–3673	990/330	**L**V**H**HA**Q**/**G**
6K1	3674–3820	147/49	**TTSS**P**E**/**S**
CI	3821–5788	1968/656	I**YHGGQ**/**E**
6K2	5789–5932	144/48	**TLIMHA**/**G**
VPg	5933–6526	594/198	**QAL**A**SE**/**T**
NIa-Pro	6527–7240	714/238	**HGA**EV**Q**/**H**
NIb	7241–8746	1506/502	**A**T**V**DG**Q**/**G**
CP	8747–9592	846/281	-
P3N-PIPO	2684–3505	824/274	-
3′-UTR	9593–9808	216/-	

**Table 2:** Recombinant analysis of SCSMV coding sequences. *p* = 0.05.

Recombinant	Major Parent	Minor Parent	Region	Detection Methods						
				R	G	B	M	C	S	T
TPT	JP2	Unknown	6602–7286	+	+	+	+	+	+	+
IND369	IND671	TPT	4122–4589	+	−	+	+	+	−	+
IND369	IND671	INDR-71	5333–6340	+	+	+	+	+	+	+
IND5268	Unknown	IND671	4818–5253	+	+	+	+	+	+	−
IND5268	INDR-71	IR-Khuz6	5667–6992	+	−	+	+	+	−	+
IND5268	IND67	YN-21	8763–9001	+	+	+	+	+	−	+
PAK	IR-Khuz6	IND671	1454–4156	+	−	+	+	+	+	+
IR-Khuz6	IR-Khua57	PAK	8318–9386	+	−	+	+	+	−	+
INDR-71	IND369	IR-Khuz6	52–774	+	−	+	+	+	+	+

**Table 3:** Recombinant analysis of *CI*, *CP*, and *P1* genes for SCSMV. *p* = 0.05.

Recombinant	Parent (Major × Minor)	Region	Detection Methods						
			R	G	B	M	C	S	T
PAK	IR-Khuz6 × IND671	CI	+	−	+	+	+	+	+
IND369	IND671 × INDR-71	CI	+	+	+	+	+	+	+
INDR-71	IND5268 × Unknown	CI	−	−	+	+	+	+	+
IND369	IND671 × MYA-Formosa	CP	+	+	+	+	+	+	+
INDR-71	IND671 × Unknown	P1	+	+	+	+	+	+	+

**Table 4:** Genetic distances within and between groups.

Group	I	II
I	0.03	0.3
II	0.3	0.10

**Table 5:** Selective pressure analysis of SCSMV. *p* = 0.1.

Protein	d_N_/d_S_	Sites under Positive Selection	Protein	d_N_/d_S_	Sites under Positive Selection
P1	0.0938	1	VPg	0.0244	0
HC-Pro	0.0476	0	NIa-Pro	0.0681	0
P3	0.0767	0	NIb	0.0367	0
6K1	0.0258	0	CP	0.0763	0
CI	0.029	0	P3N-PIPO	0.112	1
6K2	0.0013	0			

## Data Availability

Not applicable.

## Appendix A

**Table A1:** The name, sequence, amplified fragment, and annealing temperature of primers used in this study.

Primer Name	Nucleotide Sequence (5′–3′)	Amplified Fragment /nt	Annealing Temperature/°C
SCSMV-1F	AAATGTAATTTCAAATTGACTACAATCA	1–1387	55
SCSMV-1R	CATGTTCTCGTAAGCTTCGTC		
SCSMV-2F	TATACTGATGCTCATAAGGCAAG	1271–2838	55
SCSMV-2R	GACTCAGCTGTCCGATATTTG		
SCSMV-3F	ACTTGCGAAGAATGATAGCATC	2697–4203	55
SCSMV-3R	CTAGCTGCGAGGTTAATCGAT		
SCSMV-4F	TGTTATTGGTGGTGTTGGAAC	4087–5610	55
SCSMV-4R	GTGTTAAATAGGTTTGTCAACCG		
SCSMV-5F	AAGTGGATAGAACTATTGGAATCTTAC	5490–7007	55
SCSMV-5R	AAGTGGATAGAACTATTGGAATCTTAC		
SCSMV-6F	ATCATCATTCTACGTAAGTGAACAC	6895–8407	55
SCSMV-6R	CCTATGCGACATGTACTCGAC		
SCSMV-7F	ATGTACCAAGATACGCGAATC	8296–9781	55
SCSMV-7R	CCTCCTCACTGGGCAGGTTG		
SCSMV-9408F	CACCAGCAGAAATAGACGTGCGTAAC		55
Oligo(dT)_18_	CAGGATCCAAGCTTTTTTTTTTTTTTTTTT		
5′RACE-Q_T_	CCAGTGAGCAGAGTGACGAGGACTCGAGCTCAAGCTTTTTTTTTTTTTTTTT		55
5′RACE-Q_0_	CCAGTGAGCAGAGTGACG		
5′RACE-Q_I_	GAGGACTCGAGCTCAAGC		
SCSMV-413-R	CATATACTTCTGCTGCAATGTCG	1–413	
SCSMV-337-R	CTTACCTTTAACCGTCCAGAAGAC	1–337	

**Table A2:** The amino acid compositions and codon usage frequency of YN-21.

Amino Acid	Abbreviation	Codon	Number	Amino Acid	Abbreviation	Codon	Number
Alanine	Ala	GCG	27	Proline	Pro	CCG	9
		GCA	74			CCA	83
		GCU	76			CCU	24
		GCC	31			CCC	20
Cystine	Cys	UGU	29	Glutarnine	Gln	CAG	46
		UGC	22			CAA	91
Aspartic acid	Asp	GAU	112	Arginine	Arg	AGG	24
		GAC	62			AGA	40
Glutamic acid	Glu	GAG	84			CGG	19
		GAA	112			CGA	42
Phenylalanine	Phe	UUU	71			CGU	25
		UUC	64			CGC	22
Glycine	Gly	GGG	31	Serine	Ser	AGU	33
		GGA	81			AGC	32
		GGU	41			UCG	26
		GGC	17			UCA	65
Histidine	His	CAU	51			UCU	23
		CAC	50			UCC	8
Isoleucine	Ile	AUA	56	Threonine	Thr	ACG	42
		AUU	103			ACA	101
		AUC	58			ACU	53
Lysine	Lys	AAG	83			ACC	24
		AAA	96	Valine	Val	GUG	61
Leucine	Leu	UUG	63			GUA	31
		UUA	59			GUU	79
		CUG	18			GUC	26
		CUA	33	Tryptophan	Trp	UGG	49
		CUU	44	Tyrosine	Tyr	UAU	70
		CUC	47			UAC	44
Asparagine	Asn	AAU	62	Methionine	Met	AUG	84
		AAC	77				

**Table A3:** Nucleotide/amino acid identity rate between SCSMV-YN and 15 other isolates.

Isolation	Complete Genome	CDS nt/aa	P1	HC-Pro	P3	6K1	CI	6K2	VPg	NIa-Pro	NIb	CP	P3N-PIPO
JF488066	97.43	97.66/99.30	98.32/99.72	97.02/98.51	98.28/99.70	98.64/100.00	97.61/99.70	97.92/97.92	96.30/98.99	97.34/99.58	97.94/99.20	97.75/98.93	98.18/98.91
JF488065	97.42	97.65/99.30	98.32/99.72	97.02/98.30	97.98/99.39	99.32/100.00	97.36/99.54	97.92/100.00	96.97/98.99	97.34/99.58	97.94/99.40	97.98/99.29	98.42/98.91
JF488064	97.41	97.64/99.30	98.23/99.72	97.02/98.3	97.88/99.39	99.32/100.00	97.41/99.70	97.92/100.00	96.80/98.99	97.34/99.58	98.07/99.40	97.86/98.93	98.30/98.91
KJ187047	97.08	97.46/99.23	98.23/99.72	97.09/98.72	98.28/99.39	97.28/100.00	96.75/99.39	96.53/100.00	96.30/98.48	97.20/99.16	98.01/99.40	97.98/98.93	98.18/98.91
KJ187048	97.04	97.40/99.30	97.86/99.44	96.81/98.30	98.08/99.70	97.96/100.00	96.85/99.70	96.53/100.00	96.63/98.99	97.20/99.58	97.94/94.82	97.75/98.93	98.30/98.91
JN163911	96.85	97.01/99.17	96.46/98.88	96.74/98.51	97.37/99.09	98.64/100.00	96.80/99.54	98.61/100.00	95.79/98.48	96.92/99.58	97.14/99.60	98.34/98.93	97.69/98.91
KJ187049	96.57	96.86/99.20	96.55/99.16	96.60/98.51	97.07/98.79	96.60//97.96	96.44/99.70	97.22/100.00	96.46/98.48	96.92/99.16	97.01/99.80	97.98/98.58	97.57/98.91
GQ246187	94.83	96.22/98.75	96.37/99.16	96.31/98.09	97.47/98.79	80.27/100.00	97.36/99.39	97.92/100.00	95.62/98.48	90.90/97.48	96.68/99.20	94.42/97.86	98.42/98.91
KX430772	82.13	81.82/94.25	83.61/94.13	76.60/87.45	85.45/94.55	76.87/93.88	81.00/95.88	75.69/100.00	82.66/97.47	83.05/97.06	81.47/94.22	86.71/96.09	87.23/92.34
KX823354	82.01	81.72/92.59	79.87/83.38	76.45/86.26	84.65/94.55	80.27/91.84	81.71/95.58	75.69/100.00	81.48/97.98	82.52/94.12	81.54/94.42	95.10/95.73	86.50/91.97
KX823355	81.55	81.34/92.12	80.33/85.87	77.52/89.57	83.43/91.52	77.55/85.71	80.67/93.46	76.39/100.00	81.82/96.46	81.09/91.67	80.35/93.06	91.34/94.66	82.27/84.75
KX430771	81.67	81.34/94.82	83.71/95.25	76.60/89.57	85.05/94.24	74.83/91.84	80.54/96.80	74.31/100.00	81.99/97.98	83.05/97.48	80.35/94.62	85.88/94.66	86.74/92.34
GQ388116	81.60	81.25/95.11	82.31/94.97	77.80/90.00	84.95/94.85	80.27/93.88	79.67/97.41	75.69/100.00	81.14/96.97	82.63/97.06	80.61/95.02	86.12/95.37	86.25/91.24
KX823356	81.48	81.18/93.58	81.90/88.37	76.95/88.79	84.34/93.64	80.95/91.84	78.81/96.49	76.39/100.00	81.99/97.47	80.39/92.86	80.81/94.82	97.50/95.37	86.01/90.51
JN941985	81.32	80.98/87.81	81.53/87.81	77.52/90.21	84.75/95.76	80.95/91.84	79.78/97.41	75.69/100.00	80.98/97.98	81.37/94.54	80.41/94.82	86.12/96.09	86.01/90.88
